# Supplementary material for: Are carbon emissions trading and green financial instruments synergistic? -Comprehensive quantitative research based on content analysis
Source: PLoS One. 2024 Mar 7;19(3):e0298601. doi: 10.1371/journal.pone.0298601 (PMC10919601; doi:10.1371/journal.pone.0298601)
Supplement: S1 Data — (ZIP) [file pone.0298601.s001.zip › Data/地级市Z_F工作报告.zip!/╒π╜¡/╓█╔╜╩╨/└┤╫╘╣1⁄2╓┌║┼í╛┬φ┐╦╩2╛▌═°í┐_2015.docx]

各位代表：

现在，我代表市人民政府向大会报告工作，请予审议，并请政协委员和其他列席会议的同志提出意见。

一、2014年的主要工作

2014年是承上启下、全面实施新区建设三年行动计划的关键之年，也是极不平凡的一年。一年来，我们面对宏观严峻、微观复杂的经济形势，面对总量偏小、底子薄弱的客观现实，面对百舸争流、不进则退的激烈挑战，不断适应经济新常态、发展新要求，主动应对各种新情况，破解新问题，坚持稳增长促转型、打基础蓄后劲、谋创新求突破，克难攻坚，奋力拼搏，确保了经济平稳较快增长，增强了新区发展的后劲和活力。经过全市上下的共同努力，2014年地区生产总值突破1000亿元，增长10.2%；财政总收入149亿元，一般公共预算收入101亿元，分别增长8.4%和9.1%；城乡居民收入达到41466元和23783元，分别增长9.7%和11.1%；地区生产总值、固定资产投资等7项经济指标增速名列全省首位，较好地完成了市六届人大四次会议确定的目标任务。

这一年，我们强势推进新区开发建设。

——以千亿投资计划为目标的项目建设有切实成果。大力开展项目建设和招商引资年活动，实现固定资产投资961亿元，增长28.1%。大陆引水二期、浙能六横电厂、多端柔性直流输电示范工程等项目建成投用，黄泽山油品、小洋北侧围垦、浙江大学海洋学院、官山大桥等125个市级重点工程进展顺利，观音文化园、金塘重型钢管制造等95个重大项目如期开工，大陆引水三期、舟山电厂二期扩建、光汇万吨级码头等重大项目获得国家核准。实际利用市外资金402亿元，超额完成年初目标，签约引进10亿元以上产业类项目10个，其中50亿元以上项目2个。浙商回归引进项目到位资金115.8亿元，山海协作项目到位资金185亿元，分别完成年度目标任务的105.3%和308.5%。

——以船舶、水产为重点的产业转型升级有明显成效。整合提升船舶行业，对8家龙头企业和14家特色企业予以重点扶持，7家企业进入全国造船行业“白名单”，累计削减船舶产能107万载重吨，实现规模以上船舶修造业产值762亿元，增长14.4%，造船完工量、新接订单和手持订单量占全国比重分别达到11.9%、10.9%和12.6%。规范发展水产行业，铁腕整治液氨制冷的安全隐患，关停企业101家，整改提升248家，规模以上水产加工业产值增长10%。高度关注重点企业运行，出台扶持中小企业等一系列政策，防范化解企业“两链”风险，不良贷款率得到有效控制，骨干企业保持平稳运行。积极推进港航联动，全市港口货物吞吐量3.47亿吨，增长10.6%；集装箱吞吐量74.9万标箱，增长29.8%。积极发展海洋旅游新业态，国际邮轮码头建成开港，水上飞机项目和旅游观光巴士投入运营，推出了新型海岛游主题产品，共接待游客3398万人次，实现旅游收入338亿元，分别增长10.8%和12.8%。做大做强大宗商品交易平台，完成线上交易15566亿元、线下实体贸易252亿元，分别增长121%和116%。大力发展远洋渔业，远洋渔船达到450艘，产量、产值分别增长32.7%和21.7%。建成省级现代农业综合园区和特色农业精品园各1个、省级现代农业主导产业示范区2个。注重科技人才对产业转型升级的作用，引进科技创业领军人才项目21个、高端紧缺人才49名，省“百人计划”作用明显，网易舟山研发中心等项目启动，北京大学海洋研究院签约，海洋科学城核心区块建设稳步推进。

——以“四张清单一张网”为标志的重点改革有实质突破。加快政府自身改革，市级部门行政权力和审批管理事项分别精简52.7%和16.1%，政府部门权力清单和责任清单、企业投资负面清单、财政专项资金管理清单向社会公布，政务服务网投入运行。试行企业投资项目“零审批”“零收费”，全面推行商事登记“四证联办”。“大市场”监管、“大平台”检测、陆上和海上综合行政执法等管理体制改革全省领先。重组商贸集团和国有航运企业资产，成立了海域海岛使用权储备交易中心、海洋资源收储公司。新区土地利用总体规划成功获批，首期新增12万亩建设用地规划指标，7.4万亩基本农田省内统筹政策正式落地。拓宽融资渠道，债券融资达到59.3亿元。60%的村（社）完成集体资产股份合作制改革，推进新型户籍管理制度改革试点。舟山港综合保税区本岛分区实现封关运作，建立大通关模式，累计引进企业1107家，注册资金150亿元。保税燃料油供应中心建设取得实效，改革突破了一船多供、跨关区燃油直供、同税号下混合调和等政策，完成直供量66.5万吨、结算量337.2万吨，分别增长62.2%和17%，实现逆势增长。普陀山机场新增航线4条，吞吐量突破50万人次。扩大开放水陆域面积20.29平方公里，舟山获批成为全国首批进口粮食指定口岸。

这一年，我们坚持统筹协调发展。

——建设生态新区美丽海岛。以“清河净水”为抓手的“五水共治”强力推进，82条100公里垃圾河清理全面完成，70条118公里黑臭河有效整治，渔农村生活污水治理新增受益户2万余户。“三改一拆”提前完成省下达的三年任务，创建“无违建乡镇”15个。交通治堵工作全省领先，全面实行本岛公交一小时免费换乘，东山隧道复线等建成投用。舟山渔场修复暨“一打三整治”行动扎实推进，取缔涉渔“三无”船舶1067艘，整治船证不符渔船1182艘，增殖放流各类苗种4.9亿尾（粒）。生态环境保护成效明显，实行刷卡排污和排污许可证“一证式”管理，淘汰黄标车和老旧汽车7105辆，神华国华舟山电厂和浙能六横电厂成为全国超低排放的示范。“四边三化”加快推进，双桥至朱家尖生态廊道改造提升，在全省率先实现美丽乡村创建全域化。节水型社会、国家环保模范城市等创建工作通过国家部委验收和评估。

——真情关切民生社会需求。全市民生社会事业支出120.9亿元，增长13.6%，占公共财政预算支出的64.3%。十方面为民实事项目全面完成。13401名被征地农民转轨参加城镇职工养老保险，城乡居民基础养老金每月提高到125元，大病患者报销比例提高10个百分点，农村低保标准提高到城镇水平的90%，出台了原集体捕捞渔民生活补贴政策。加强“菜篮子”保障，改造提升农贸市场6家，新增市级蔬菜基地2075亩，累计达到16000亩。推进社会事业投入多元化，出台了扶持民办教育、医疗和养老机构的政策意见，上海瑞金医院舟山分院签约落户。实现义务教育基本均衡化创建目标，支持浙江海洋学院创建海洋类大学，新区旅游与健康职业学院（筹）正式开学，舟山技师学院完成主体工程。公共卫生服务项目扎实推进，公立医院综合改革进一步深入，省卫生强市顺利通过考核验收。出生人口素质不断提升，在全省率先实施“单独两孩”政策。创新打造“淘文化”平台，新建成文化礼堂44家，舟山博物馆、海洋数字图书馆、网上方志馆等投入运行，市民大讲堂、公民素质讲习所等作用日益明显。精神文明创建活动深入开展，我市被推荐为全国文明城市提名城市。市体育场动工建设，学校体育场地设施向社会有序开放。深入推进军民融合发展示范区建设，人民防空、海防与反走私等工作扎实开展，第八次荣膺“省双拥模范城市”。深化平安舟山建设，组织打防侵财犯罪等专项行动，加强社会治安防控体系建设，群众安全感满意率居全省前列。加强安全生产、公共安全和气象灾害防御，实施油库管线、水路货运船舶安全专项整治，安全生产事故起数、死亡人数连续十年实现零增长。认真做好信访工作，民族、宗教、侨务、档案事业不断发展，工青妇等群团组织作用进一步发挥，儿童、老龄、红十字、慈善和残疾人事业等取得新成绩。

——切实加强政府自身建设。认真学习贯彻习近平总书记系列重要讲话精神，深入开展党的群众路线教育实践活动，75项民生即知即改项目全面完成，“四风”问题得到有力整改。自觉接受人大、政协监督，定期向人大报告工作、向政协通报情况，共办复各级人大代表议案建议149件、政协提案344件。重视社会舆论监督，开展“电视问政”“网络问政”。发挥新区决策咨询委作用，建立了新区研究院，聘请了10名新区发展顾问，加强与专业机构的研究合作，努力提高决策科学化水平。加强政府性投资项目规范管理，设立公共项目建设中心，政府投资项目造价中心和审计中心作用明显。强化审计监督，核减全市政府投资项目支出4.96亿元。从紧控制行政成本，全市“三公”经费支出减少32.9%。提高财政专项资金使用效率，市本级集中财政专项资金1.08亿元，专项用于“五水共治”。政务云平台一期建成投用，市级部门信息化资源得到有效整合。加强市本级行政事业单位房屋土地资产集中管理。做好办公用房清理规范工作，腾退办公用房5.75万平方米。开展正风肃纪和窗口单位服务行业专项整治，共查处各类违规违纪人员313人。

各位代表，过去一年的成绩来之不易。这是省委省政府和市委正确领导、市人大和市政协监督支持、全市百万军民共同奋斗的结果。在此，我谨代表市人民政府，向各位人大代表、政协委员，向全市人民和建设者，向驻舟部队和省部属在舟单位，向所有关心支持舟山发展的社会各界人士和海内外朋友们，表示衷心的感谢和崇高的敬意！

回顾一年来的工作，我们也有遗憾和不足：舟山自由贸易港区虽经全力争取并得到国家的基本认可，但由于国家实施对外开放战略新布局，自贸港区未能成功获批，需要我们坚持不懈地谋划申报；受宏观经济下行和国际油价持续下跌等影响，外贸出口持续负增长，没有完成年度目标任务；一些企业生产经营仍面临不少困难，重大项目引进不多、建设进度不快，新的经济增长点不明显，财政收支平衡压力较大，新区可持续发展能力亟待提高；社会保障、公共服务和食品安全等与群众要求还有不少距离，富民惠民力度还需不断加大；公务员队伍思想观念还不够解放，担当精神还不足，破解新问题的办法和举措还不多；官僚主义、形式主义和消极腐败现象仍然存在，反“四风”的长效机制有待继续巩固。对于这些问题，我们将认真研究，努力解决。

二、2015年的总体要求和中心工作

今年是实施新区建设三年行动计划的冲刺之年，是“十二五”规划的收官之年，也是“十三五”规划的谋划之年。纵观今年的形势，对舟山群岛新区来说，机遇与挑战并存，希望与困难同在。一方面，我国经济发展已进入“保持中高速增长、迈向中高端水平”的新常态，给新区开发建设带来了前所未有的新挑战。另一方面，舟山作为国家建设“21世纪海上丝绸之路”和“长江经济带”的战略支点，将迎来千载难逢的新机遇。近期，李克强总理明确要求“加快建设舟山江海联运服务中心”。这是总理对舟山群岛新区的殷切期望，是在舟山自由贸易港区谋划申报的基础上，国家赋予我们的又一新的战略任务，也是新区大开发大建设的重大举措和重要支撑。我们必须清醒把握形势，抢抓发展机遇，勇担历史使命，在经济新常态下努力彰显新作为，实现新突破！

今年工作的总体要求：深入贯彻党的十八大、十八届三中、四中全会和习近平总书记系列重要讲话精神，全面落实中央、省委省政府和市委的决策部署，继续坚持“稳中求进、进中求快、快中求好”的总基调，把实施国家战略作为最大的政治任务，把加快新区发展作为最重要的政治纪律，把谋划建设舟山江海联运服务中心作为“一号工程”，精心编制“十三五”规划，进一步突出转型升级、改革攻坚、创新驱动、民生改善、依法治理，大干实干苦干巧干，全面加快新区发展。

建议今年经济社会发展的主要预期指标为：地区生产总值增长9%以上，固定资产投资增长20%，一般公共预算收入增长8%，城乡居民收入分别增长9%和10%，单位生产总值能耗及化学需氧量、二氧化硫等节能减排指标完成省定的目标。

今年的中心工作是：全力抓好舟山江海联运服务中心建设“一号工程”，并以此统领突破新区全方位的开发建设，加快形成新区的综合竞争力。

实施“一号工程”，需要解放思想，树立新理念。舟山江海联运服务中心建设，这是一个全新的课题。要求我们坚持先行先试，以更宽的视野、更高的层次、更大的格局探索江海联运新模式，丰富现代服务新理念，奠定舟山江海联运服务中心的新地位；坚持全面开放，发挥舟山通江达海的独特区位优势，更好地利用国际国内两个市场、两种资源，构建舟山江海联运服务中心对外开放新格局；坚持区域联动，主动处理好与上海、宁波等周边沿海城市，以及武汉、南京等长江沿线城市的关系，促进港口合理布局、产业合理分工、要素合理配置，实现互利多赢；坚持生态和谐，突出绿色生态理念，建立严格的生态环境保护制度，集约节约开发利用海洋和海岛资源，建设海上花园城。

实施“一号工程”，需要突出重点，明晰新路径。舟山江海联运服务中心建设，涉及面广、内涵丰富。要求我们重点建设我国最为完善的江海联运综合枢纽，打造集装箱转运基地，布局建设长江内支线集装箱码头和1.8万标箱及以上的大型集装箱泊位，加快拓展宁波-舟山港集装箱码头的发展空间，保障长江经济带集装箱的江海中转和水陆中转，提高与周边国际港口的竞争水平；打造大宗散货集散中心，以原油、矿砂、煤炭、粮油和化工品等大宗物资为主，重点建设一批专用泊位、减载平台、分拨堆场、保税仓库等集散设施，降低长江沿线、内陆腹地发展所需的大宗物资运输成本，提高区域经济的综合竞争力。建设我国最大规模的大宗商品储备加工交易基地，增加国家原油战略储备与商业储备，扩大铁矿砂贸易储运园区和保税堆场，建设一批粮油、钢材、有色金属、煤炭、木材等大宗商品的储备基地、港航物流园区。积极规划建设粮油加工基地、绿色石化基地，保障国家战略资源需求和经济安全。加快大宗商品交易中心建设,研究设立期货交易所，争取组建国际大宗商品交易所，开展保税期货交易，切实服务长江经济带发展。建设我国现代示范的江海联运服务体系，加快发展以保税燃料油加注为核心的国际海事服务，创新和丰富现代海事服务内容，培育和拓展服务新业态；集聚发展江海联运服务产业，建设江海联运服务总部，提升江海联运信息化水平，建设江海联运大数据平台；打造江海联运船队，发展江海直达、沿海和国际航运等三大运力，增强我国国际海运市场竞争力。同时，我们还要切实加强要素保障和制度创新，强化能源、供水、用地、用海等基础性支撑，打造多式联运、便捷高效的综合交通网络，完善对外开放、行政管理、区域合作等方面体制机制，为舟山江海联运服务中心建设营造良好的环境。

实施“一号工程”，需要立说立行，实现新突破。舟山江海联运服务中心建设，前景十分广阔，但挑战也非常严峻。要求我们只争朝夕、狠抓当前。一是全力争取舟山江海联运服务中心建设列入长江经济带发展规划，早日获批建设方案，落实支持政策。二是推进金塘大浦口集装箱项目建设，力促鼠浪湖矿砂、光汇油品、黄泽山油品等项目建成投产，加快华泰石油储运中转、册子实华二期等项目进度，推进马迹山三期、六横煤炭二期等项目前期工作；创新合作机制，推动小洋山北侧加快开发，谋划衢山、六横、大洋山等港区的开发时序和模式。三是根据国家战略规划，在实行最严格的环保准入和监管措施的前提下，科学评估，选择在合适的区域发展绿色生态、国际一流的石化产业；加快建设舟山国际海事服务基地、国家远洋渔业基地和国际粮油产业园区。四是建立航运发展基金，组建壮大江海联运船队，促进航运业稳定发展和做大做强；建设江海联运公共信息平台、航运电商物流服务平台。五是建成运行浙江石油化工交易中心，做大做强煤炭、粮食、远洋水产品和石油化工等特色交易，积极争取舟山大宗商品交易中心获国家授牌。六是推进六横公路大桥建设相关工作，开工建设富翅门大桥，加快秀山大桥等工程建设，全力争取舟山本岛-岱山-洋山-上海的北向大通道和甬舟铁路列入国家“十三五”规划，推进普陀山机场的扩能改造和空港开放，推进蛇移门航道、条帚门外锚地等建设。

三、2015年的工作任务

围绕总体要求、主要目标和中心工作，今年还要认真抓好以下七方面工作。

（一）致力打造法治政府。坚持法定职责必须为、法无授权不可为，完善“四张清单一张网”，推进政府机构、职能、权限、程序、责任法定化。深化行政审批制度改革，全面实行“零地”项目政府不再审批、企业独立选址项目高效审批，建立健全事中事后监管制度，全面取消非行政许可审批事项。提升行政执法规范化水平，深化海洋综合行政执法改革试点，健全综合执法协调配合机制。完善重大行政决策程序，健全重大行政决策听证、社会稳定风险评估、合法性审查和向人大报告制度，全面推行政府法律顾问制度，规范政府合同管理。稳步推进公共管理市场化改革，完善政府购买公共服务目录和实施办法，探索公共服务供给主体多元化。加强公共资源交易、政府项目投资、财政资金使用等重点领域和关键环节的监管。深化国企国资改革，鼓励支持民间资本参与国企改革，发展混合所有制经济。深化政府投融资体制改革，做大做强海投、交投等国资平台，加快舟山港股份上市，提高直接融资比重。积极引进各类金融机构，鼓励发展融资租赁业。

（二）加快产业转型升级。坚持育增量、优存量、扩总量，继续把招商引资作为新区发展的“生命线”，实行全员招商、专业招商、精准招商，发挥经济功能区的主战场、主力军作用，引导产业和项目向园区集聚。进一步加强浙商回归、激发民资、引进外资、对接央企等工作，主动服务、全力推进项目快落地、快建设、快投产，提高招商引资的质量和贡献度。坚持实施创新驱动战略，推进海洋科学城建设，力促北斗及海洋通信应用、北京大学海洋研究院等项目加快建设。深入实施“四换三名”工程，加快建立以企业为主体的技术创新体系，研究制定促进科技成果转化的政策，力争技改投入达到150亿元，增长10%。重视人才队伍建设，优化人才环境，鼓励人才创业特别是青年创业。坚持工业主导地位，推进工业化与信息化深度融合，发挥船企“白名单”效应，促进船舶制造业健康发展。启动航空产业园建设，全力争取重大项目落户。建设海洋数据中心，培育信息经济，促进电子商务发展。积极发展海洋工程装备、海洋生物医药、海洋新能源等产业。扶持建筑业做大做强。高度重视实体经济发展，加强企业帮扶，严密防控企业“两链”风险。坚持海洋旅游业提升发展，申办首届世界海岛旅游大会，推进普陀山景区全面提质，加快观音文化园建设，培育邮轮游艇、养生健康等产业，建设特色小镇（街区、乡村），完善旅游基础设施，打造群岛型海洋休闲旅游度假目的地。坚持渔农业基础地位，培育新型经营主体，促进规模经营、特色发展。加快建设国家远洋渔业基地，建成远洋渔业专用码头、专业市场，延伸远洋水产品产业链，完善远洋渔业综合服务保障功能。发展休闲渔农业，推进渔港经济区建设。

（三）扩大新区对外开放。全力打造国际海事服务基地，继续争取各级支持，创新监管方式，力争保税燃料油直供量达到150万吨、结算量超过500万吨，均增长50%以上，跻身全国前三位；着力拓展海事服务领域，组建外轮供应服务中心，提供船舶维修、货运代理、淡水和物资补给等服务；开展国际二手船舶（游艇）的保税交易。继续加强舟山自由贸易港区的申报工作，推进综保区探索试行自由贸易港区政策，加快衢山分区建设，力促“海外仓”项目及早落地，打造进出口商品集散中心，提升综保区辐射作用。做好综保区配套码头、国家远洋渔业基地专用码头等口岸开放工作。完善大通关服务，建立货物分类监管模式。提升对外贸易水平，力争实现外贸进出口132亿美元，其中出口62亿美元，均增长7.5%。鼓励企业“走出去”，开展对外经济合作。加强与宁波、上海及长江沿岸城市的交流互动，主动融入长三角。推进与港澳台、国外友好城市的经贸文化合作交流，加快浙台经贸合作区建设，争取对台“自由行”早日获批。

（四）加快城乡统筹发展。强化城乡一体化发展和本岛全域城市化的理念，集聚资源要素，逐步统一规划、建设和管理。启动百里滨海大道建设，打造生态景观、历史文化、民生休闲、产城提升的长廊。加快完善新城城市功能，发展现代服务业和都市型产业，进一步形成集聚效应。加快老旧城区改造，重点推进定海和平路区块和普陀半升洞区块改造提升。提升美丽县城建设水平，加快金塘、六横小城市培育和中心镇发展。深化“三改一拆”，全面推进“无违建县（区）”创建，完成6个城中村综合改造试点，建设美丽海岛综合示范区块和精品社区。推进“交通治堵”，构建智慧公交系统。加快城乡综合配套改革，全面完成渔农村集体资产股份合作制改革，深化户籍制度改革，加快推进渔农村宅基地、集体建设用地的使用权确权和退出使用工作。加快推进大陆引水三期和嵊泗大陆引水工程，继续谋划实施海水淡化项目。加快推进500KV电力联网工程前期工作，实施220KV岱山输电改造和本岛城区配电网改造提升。加快329国道舟山段改建，建成定马公路复线，推进岛际交通资源整合。积极推进岸线整理和收储，加快实施钓梁三期、岱山北部和大小鱼山等围垦促淤工程。

（五）推进生态文明建设。扎实推进“五水共治”，确保黑臭河治理全面达标。加快本岛污水处理厂能力提升及污水管网建设，完成建制镇（街道）污水处理设施建设，改革污水处理管理体制。实施渔农村生活污水治理扩面改造工程，确保新增受益渔农户4万户以上。加强饮用水水源地环境保护，抓好渔农村饮水安全提升工程，新增受益渔农民2.5万人。继续推进生态廊道工程，加快平原绿化和湿地公园建设。推进耕田垦造，建立低效用地企业退出机制，推行地下空间有偿使用。实施舟山渔场修复振兴工程，全面完成省下达的“一打三整治”三年任务。坚持保护与开发并重，加强对禁止开发区、限制开发区的生态保护，严格执行项目环保评估一票否决制。推进国家海洋公园、中街山列岛等海洋保护区建设，加强近岸海域污染整治。强化节能减排刚性约束，全市燃煤锅炉和工业窑炉基本完成脱硫除尘设施改造。加快永久性基本农田土壤治理，推进危废填埋场、污水处理厂污泥处置等项目建设，加强畜禽养殖场污染治理，实行病死动物统一处理，整治重污染行业。全面创建国家生态文明示范区，巩固国家环保模范城市创建成果，争取通过国家卫生城市首次复审。

（六）深化平安舟山建设。推进基层平安创建活动，强化普法教育，完善矛盾纠纷大调解机制，规范信访工作秩序，正确引导网络舆论。完善基层社会治理机制，深化“网格化管理、组团式服务”，推进便民服务事项进社区，加强流动人口服务管理，构建和谐劳动关系，提高为民服务水平。严格落实安全生产责任制，积极实施安全生产标准化，加强海上生产、油品储运、无动力船舶、宗教场所、跨海大桥等安全管理，深入开展水路货运船舶安全专项整治。强化各类应急培训，建设突发公共事件预警信息发布平台，构建基层气象防灾减灾体系。完善立体化社会治安防控体系，依法严厉打击各类违法犯罪活动，健全反恐防范体系，营造良好的新区发展环境。

（七）全面促进富民惠民。落实好政府“保基本、守底线”的政治责任，进一步加大民生社会事业的投入，让人民群众共享新区发展成果。大力推进就业创业，以创业推进高质量就业，加快大学生创业园建设，重点做好高校毕业生的就业创业工作。强化渔农民素质培训，促进渔农民增收。新增城镇就业1万人，城镇登记失业率控制在4%以内。扎实做好社会保障工作，推进社会保险的全覆盖和逐步并轨，实施机关事业单位工作人员养老保险制度改革，做好被征地农民养老保险转轨工作，实施原集体捕捞渔民生活补贴政策。完善养老服务保障体系，大力推进居家养老，鼓励社会力量参与养老事业。大力弘扬慈爱文化，加强扶贫救助体系建设，继续做好低收入户脱贫帮困工作，全面推进残疾人康复救助。继续加强菜篮子工作，加快城乡农贸市场提升改造，推进畜禽定点屠宰，努力平抑菜价，实现蔬菜、水产品直供门店本岛全覆盖。办好人民满意教育，加快教育改革发展，推进学前教育公益普惠化发展，高标准推进义务教育均衡化，确保浙江大学海洋学院如期开学、新区旅游与健康职业学院顺利摘筹、舟山技师学院工程建成投用，推进浙江海洋学院创建海洋类大学。更好地满足群众健康需求，深化公立医院改革，推进市级优质医疗资源下沉，构建科学合理的分级诊疗机制，完善基层医疗卫生机构运行新机制，深化拓展基本公共卫生服务，建设“海洋健康城市”。落实计划生育政策，促进人口均衡发展。完善公共文化体育服务体系，继续推进文化礼堂建设，升级打造“淘文化”平台，办好中国海洋文化节，加快市体育场建设进度。积极推进国有经营性文化单位改革，促进文化体育产业发展。加快城乡社区电子商务配送公共服务平台建设，建成E邮站150个以上，基本实现全市城镇公共服务区域免费WIFI全覆盖。推进军民融合示范区建设，争取蝉联全国双拥模范城。扎实推进全国文明城市创建活动。

今年，我们要继续办好十方面为民实事项目：1.大力推进“五水共治”。继续实施“清河净水”工程，完成治理黑臭河43条65公里，整治河道40公里，完成90个行政村的渔农村生活污水治理；基本建成定海污水处理厂三期和小干污水处理厂二期工程，完成城乡污水管网铺设40公里。2.加快“菜篮子”工程建设。新增蔬菜基地2000亩，累计达到18000亩；创建省级放心农贸市场6家,新增菜篮子直供店和生产基地直销店（点）各10家。3.加强食品安全监管。全市90家大型、特大型餐饮企业和学校食堂建成“阳光厨房”，全面完成“千万学生饮食放心工程”，全市学校大宗食品统一配送或定点采购率达到90%。4.推进安居工程建设。新开工各类保障性住房14500套，竣工7300套，交付4200套。5.强化社会保障和帮扶。全面实现城乡低保标准一体化，城乡最低生活保障标准提高至588元，新增养老床位1000张，为全市60%的80周岁以上老年人配备多功能“一键通”应急呼叫终端；为1500名残疾人实施助明、助听、助行及残疾儿童抢救性康复服务。6.持续治理大气污染。全面完成黄标车淘汰任务；完成中海油舟山石化有限公司脱硫和脱硝改造、旺能环保能源有限公司烟气脱硝等工程，保持优良空气质量。7.深化交通惠民。建成盐仓、岑港两个公交枢纽站和150个港湾式停靠站，新增公交车40辆；完成城市绿道建设10公里；建成出租车“电召”服务管理信息系统。8.加快文体设施建设。举办百团百艺进文化礼堂活动200场、市民大舞台40场；全市所有村完成应急广播体系建设。9.促进教育公平。义务教育标准化学校比例提高到80%，普惠性幼儿园覆盖面达到75%以上，等级幼儿园覆盖面达到85%以上；全市公办义务教育阶段学校建成直饮水工程。10.加大医疗惠民力度。启动市二院迁建工程，市中心血站完成主体工程建设，开展渔农民流动医院免费巡回医疗服务50次以上，惠及群众5万人次。

各位代表，雄关漫道真如铁，而今迈步从头越。做好今年工作，我们必须振奋精神、勇担使命。以时不我待的紧迫感，舍我其谁的责任感，积极实施国家战略，认真学习借鉴外地先进经验，勇于担当，敢于破难，始终保持满腔激情，夙夜在公，勤勉工作。必须凝心聚力、同舟共济。认真落实市人大及其常委会各项决议决定，积极支持政协履行政治协商、民主监督、参政议政职能，广泛听取民主党派、人民团体和各界人士的意见建议，自觉接受各方面的监督，集思广益，群策群力。必须从严从实、廉洁奉公。巩固和发展党的群众路线教育实践活动成果，严明政治纪律，严守八项规定，加强“四风”突出问题整改，完善预决算制度，“三公”经费只减不增，完成公务用车改革，切实加强制度建设，坚决反对腐败、惩治腐败。

各位代表，机遇成就梦想，实干铸就辉煌。让我们更加紧密地团结在以习近平同志为总书记的党中央周围，在省委、省政府和市委的坚强领导下，紧紧依靠全市人民，同心同德，四干兴舟，为不断开创浙江舟山群岛新区建设的新局面而努力奋斗！
